# Leveraging Single Nucleotide Polymorphism Profiling for Precision Skin Care: How SNPs Shape Individual Responses in Cosmetic Dermatology

**DOI:** 10.1111/jocd.16750

**Published:** 2024-12-31

**Authors:** Diala Haykal

**Affiliations:** ^1^ Centre Médical Laser Palaiseau Palaiseau France

**Keywords:** cosmetic dermatology, genetic predisposition, personalized skincare, precision medicine, single nucleotide polymorphisms (SNPs) profiling

## Abstract

**Introduction:**

Single‐nucleotide polymorphisms (SNPs) represent a significant genetic variation influencing individual responses to cosmetic dermatology treatments. SNP profiling offers a pathway to personalized skincare by enabling practitioners to predict patient outcomes, customize interventions, and mitigate risks.

**Background:**

The integration of genetic insights into dermatology has gained traction, with SNP analysis revealing predispositions in skin characteristics, such as collagen degradation, pigmentation, and inflammatory responses. Key SNPs, including MMP1, SOD2, TYR, and IL‐6, are pivotal in determining skin health and treatment outcomes. Despite its promise, the adoption of SNP profiling in cosmetic dermatology is in its infancy, requiring further exploration of its practical applications.

**Results:**

SNPs significantly influence skin responses to aesthetic treatments, offering insights for personalized care. Variations in MMP1 correlate with collagen degradation, suggesting collagen‐stimulating therapies, while SOD2 SNPs highlight the need for antioxidant support. TYR variations affect pigmentation risks in light‐based treatments, and IL‐6 SNPs reveal inflammatory predispositions, guiding anti‐inflammatory protocols. AI integration enhances SNP profiling by improving prediction accuracy and treatment customization. Challenges remain, including standardization, ethical considerations, and cost‐effectiveness. Combining genetic insights with epigenetics and leveraging AI technologies can amplify precision and safety in dermatologic care.

**Conclusion:**

SNP profiling marks a transformative step toward precision medicine in cosmetic dermatology, enabling tailored treatments that enhance efficacy and minimize adverse effects. Integrating AI‐driven SNP analysis with epigenetic insights provides a comprehensive approach to patient care, fostering a new era of personalized skincare that respects genetic and environmental interactions. This paradigm shift holds the potential to redefine dermatologic practices, improving outcomes and patient satisfaction.

## 1 Introduction

The field of personalized medicine has introduced significant advancements in the patient‐centered care, especially in cosmetic dermatology. At the forefront of this movement are single nucleotide polymorphisms (SNPs), small genetic variations in DNA that affect how individuals respond to environmental factors, skincare products and cosmetic interventions. SNPs provide a unique window into our genetic makeup, revealing predispositions that influence skin characteristics such as pigmentation, collagen production, elasticity, and inflammation [1, 2]. These genetic insights are essential in creating a precise approach to cosmetic dermatology, tailoring treatments to the individual's genetic blueprint to optimize efficacy and safety. In this commentary, I explore the potential of SNP profiling in enhancing treatment outcomes in cosmetic dermatology and examine several key SNPs contributing to the precise cosmetic dermatological procedures.

## 2 The Role of SNPs in Skin Characteristics and Aging

Single nucleotide polymorphisms are the most common type of genetic variation among humans. These alterations occur when a single nucleotide undergoes substitution. While many SNPs have minimal effects on bodily functions, some directly influence gene expression and the biological pathways that shape our skin structure and behavior [3, 4]. Variations in SNPs can account for differences in how skin ages, responds to oxidative stress, and heals posttreatment. Cosmetic dermatology has long recognized the importance of factors such as sun exposure, diet, and skincare routines in maintaining skin health. However, the inclusion of genetic profiling, specifically SNP analysis, adds a novel dimension. By examining these SNPs, dermatologists can better predict how a patient's skin will respond to treatments, guiding more effective strategies for antiaging, pigmentation, and other skin concerns. As a result, SNP profiling can support more precise and personalized treatment plans, marking a paradigm shift in cosmetic dermatology [5].

## 3 Key SNPs Impacting Cosmetic Dermatology Treatments

One of the most promising areas of SNP application in cosmetic dermatology is assessing a patient's potential for collagen degradation and elasticity loss. For example, specific SNPs within the matrix metalloproteinase‐1 (MMP1) gene, responsible for coding the enzyme MMP1, influence the rate of collagen breakdown. MMP1 is an essential enzyme in collagen remodeling, particularly as the skin ages. Variants of MMP1 linked to higher enzyme activity can predispose individuals to accelerated collagen breakdown, resulting in reduced skin elasticity and increased wrinkle formation. With this knowledge, dermatologists can recommend collagen‐supporting treatments, such as radiofrequency or laser‐based procedures, to patients with high‐collagen degradation SNP profiles in order for the treatments to align with the skin's genetic needs [6].

Another SNP of clinical relevance is found in the SOD2 gene, which encodes superoxide dismutase, a powerful antioxidant enzyme that mitigates oxidative damage by neutralizing free radicals. Variants within the SOD2 gene can impair antioxidant defenses, making individuals more susceptible to a primary driver of skin aging, namely oxidative stress. For patients with reduced superoxide dismutase activity due to SNP variations, cosmetic dermatologists can proactively introduce antioxidant‐rich skincare regimens and recommend treatments that minimize oxidative stress. In laser therapy, for example, knowing a patient's SOD2 profile can guide the application of posttreatment antioxidants to optimize recovery and protect against further damage [7].

The tyrosinase gene (TYR), essential in melanin synthesis, is another SNP that holds significant implications for cosmetic dermatology. Variations in TYR affect pigmentation tendencies, leading to differences in how individuals respond to light‐based treatments, such as lasers or intense pulsed light therapies. For those with TYR variations linked to heightened melanin activity, there may be an increased risk of post‐inflammatory hyperpigmentation (PIH) after certain treatments. Recognizing these risks, practitioners can tailor treatments by adjusting the intensity or type of laser used and a post‐care rich in brightening formulas to ensure safer outcomes for patients prone to pigmentation issues [8].

Lastly, the IL‐6 gene, encoding interleukin‐6, is integral to immune regulation and inflammatory responses, with its expression influenced not only by genetic variants, but also by epigenetic modifications, such as DNA methylation. Variations in IL‐6 can affect cytokine expression levels, potentially modulating inflammation through interactions with chromatin structure and gene regulation pathways. These mechanisms suggest that IL‐6 SNPs, alongside epigenetic factors, may alter skin inflammation and response to cosmetic treatments [9, 10]. By understanding both genetic predispositions and epigenetic influences on IL‐6, cosmetic dermatologists can tailor interventions to better manage inflammatory responses, thus enhancing treatment safety and efficacy [11].

## 4 Future Prospects for SNP‐Based Personalization in Cosmetic Dermatology

SNP profiling provides more than just genetic insight; it opens a pathway to optimize aesthetic outcomes in cosmetic dermatology by allowing practitioners to understand each patient's unique genetic predispositions and design treatment plans accordingly [12, 13]. For example, a patient with a high risk of collagen degradation based on their MMP1 SNP profile may benefit from collagen‐stimulating treatments such as fractional lasers, microneedling, or radiofrequency, which counteract MMP1‐related collagen breakdown, ultimately improving skin elasticity and firmness. SNP profiling also guides posttreatment care, as patients with SOD2 SNP variants linked to reduced antioxidant capacity might need antioxidant‐rich regimens to protect against oxidative stress following laser procedures [14, 15]. Similarly, patients with TYR variants associated with pigmentation tendencies can be advised to avoid high‐intensity treatments and incorporate targeted skincare products that support pigmentation balance [16]. Furthermore, understanding the genetic foundations behind diverse skin responses to environmental stress factors across ethnic skin types provides insights into how SNPs influence skin aging, pigmentation, and oxidative stress responses. Relevantly, using in silico modeling and gene network analysis alongside in vitro tests, researchers identified skin biomarkers impacted by SNPs and examined their interactions in European, Asian, and African skin. These findings support the development of personalized skincare, demonstrating how targeted ingredients like resveratrol and quercetin can modulate SNP‐affected pathways, paving the way for precision‐based cosmetic formulations that cater to the genetic and ethnic uniqueness of individuals [12].

As SNP profiling technology becomes increasingly accessible and affordable, its routine use in dermatologic assessments could revolutionize patient care by refining treatment protocols based on individual risk profiles and genetic predispositions. This individualized approach not only enhances treatment efficacy, but also anticipates potential risks, providing a more preventive, proactive framework for care. Combining SNP analysis with epigenetic insights will further enrich dermatologic practice by revealing how lifestyle, diet, environmental factors, and treatments interact with genetic predispositions. Consequently, practitioners can design personalized interventions that modify gene expression through targeted lifestyle and skincare recommendations, moving beyond a one‐size‐fits‐all approach to deliver tailored, safe, and effective results that respect each patient's unique genetic makeup, at the same time maintaining a holistic approach to the patient's dermatological care [17, 18].

## 5 The Role of Artificial Intelligence in SNP Profiling and Personalized Dermatology

The integration of artificial intelligence (AI) into SNP profiling promises to accelerate the evolution of personalized medicine in cosmetic dermatology. AI algorithms can analyze extensive genomic datasets, uncovering complex relationships between SNPs and skin health that may be either vague or time‐consuming through traditional analysis. For instance, deep learning techniques can identify patterns among multiple SNPs associated with collagen synthesis, pigmentation, and inflammatory responses, helping practitioners predict individual responses to aesthetic treatments with remarkable precision. By incorporating AI‐driven insights, dermatologists can deliver even more nuanced treatment plans that account for the interplay of multiple genetic factors, advancing the field of personalized skincare. Moreover, AI‐powered diagnostic tools can evolve as new SNP‐related data emerges, making them invaluable for ongoing research and continual refinement of personalized dermatological practices [19, 20].

## 6 Discussion

The application of SNP profiling in cosmetic dermatology has the potential to redefine patient care by enabling truly personalized approaches. Unlike traditional treatments, which often adopt a standardized protocol for skin conditions, SNP profiling allows dermatologists to identify specific genetic variations that affect how a patient's skin may respond to various interventions. By understanding these genetic markers, dermatologists can personalize treatment plans to address individual skin health needs, maximizing therapeutic outcomes while minimizing risks. A key benefit of SNP profiling is its ability to enhance precision in aesthetic interventions. For example, patients with genetic variations that predispose them to increased collagen degradation, such as certain MMP1 SNPs, may benefit more from collagen‐stimulating treatments like radiofrequency or laser therapies. Conversely, patients without such predispositions might receive optimal results from treatments focusing on other skin qualities, such as hydration or pigmentation. This precision allows for more effective resource utilization and less trial‐and‐error, ultimately improving both clinical outcomes and patient satisfaction. Another area where SNP profiling shows promise is in managing adverse effects associated with cosmetic procedures. For instance, variations in the SOD2 gene can predispose individuals to oxidative stress following certain treatments. Patients with these variations may be more vulnerable to posttreatment inflammation and tissue damage. By proactively identifying these at‐risk patients, dermatologists can introduce antioxidant‐rich skincare regimens pre‐ and posttreatment, significantly reducing the likelihood of complications. Similarly, SNPs affecting pigmentation, such as those in the TYR gene, can inform practitioners about a patient's likelihood of developing PIH after laser procedures, guiding decisions about treatment selection and intensity. Moreover, SNP profiling can be a valuable tool for selecting patients who would benefit from preventive treatments tailored to their genetic makeup. For example, patients with a predisposition to inflammatory responses, due to variations in genes like IL‐6, could be guided toward anti‐inflammatory protocols, both pre‐ and posttreatment, to maintain skin stability and enhance the longevity of results. This preemptive approach may also improve treatment efficiency and outcomes for those with complex dermatologic conditions such as rosacea or chronic acne, which are often exacerbated by inflammation.

The broader implications of integrating SNP profiling into dermatology are substantial. As this technology becomes more accessible, its potential applications extend beyond treatment personalization to preventive care. Dermatologists could implement SNP screening as part of initial patient consultations, using it to educate patients about their own skin predispositions and guide them in preventive skincare. With increased understanding, patients could adopt lifestyle adjustments, dietary habits, and skincare routines aligned with their genetic profile, further enhancing skin health and resilience over time. However, there are challenges to address. For SNP profiling to reach its full potential in cosmetic dermatology, further research is needed to identify additional SNPs with specific relevance to skin health and treatment response, besides its cost‐effectiveness when integrated into dermatological procedures. Additionally, as the field of genetics evolves, there is a need to establish standardized protocols for integrating SNP analysis into practice to ensure consistent application and interpretation. Another consideration is the ethical implications of genetic profiling, particularly in maintaining patient confidentiality and ensuring informed consent. Education will also be essential, both for practitioners to stay informed on the latest genetic insights and for patients to understand how SNP profiling benefits their treatment journey.

## 7 Conclusion

The SNP profiling represents an exciting leap toward fully personalized care in cosmetic dermatology, aimed at tailoring treatments to each patient's unique genetic background. As our understanding of SNPs and their influence on skin health continues to grow, the promise of personalized skincare, thus successful treatments and patient satisfaction, is steadily becoming a reality. The incorporation of genetic profiling into aesthetic practices empowers practitioners to offer more targeted, effective, and safer treatments, setting new standards for individualized care. In addition, the future of SNP profiling could involve the integration of epigenetic insights, combining genetic predispositions with modifiable factors such as lifestyle and environmental exposure. This synergy could customize treatments that address both genetic and epigenetic influences on skin health. Furthermore, AI will play an essential role in SNP analysis, enabling comprehensive care strategies at an unprecedented scale. As the field of cosmetic dermatology moves toward this precision‐based model, SNP profiling stands out as a foundational tool, equipping practitioners to deliver results that meet the highest standards of patient‐centered care, transforming their outcomes and the way we understand and approach dermatological procedures.

## Ethics Statement

The author has nothing to report.

## Conflicts of Interest

The author declares no conflicts of interest.

## Data Availability

The data that support the findings of this study are available in the supporting information of this article.
